# Crime scene examiners' mental health: a scoping review

**DOI:** 10.3389/fpsyg.2025.1724579

**Published:** 2026-01-13

**Authors:** Zohra Ben Salah, Tony Mickelsson Blomqvist, Mehdi Ghazinour

**Affiliations:** 1 Department of Epidemiology and Global Health, Umeå University, Umeå, Sweden; 2 Department of Police Science, School of Police Studies, Södertörn University, Huddinge, Sweden

**Keywords:** anxiety, coping, crime scene investigator, depression, mental health, PTSD, resilience

## Abstract

Crime scene examiners (CSEs) play a crucial yet understudied role in law enforcement, frequently exposed to traumatic material under demanding organizational conditions. While the psychological health of frontline police has been extensively investigated, little is known about the mental wellbeing of those working in forensic and crime scene units. This scoping review systematically maps the existing international literature on CSEs' mental health to identify key themes, knowledge gaps, and implications for practice. Following Arksey and O'Malley's (2005) framework and PRISMA-ScR guidelines, seven databases were searched, yielding 24 studies published between 2005 and 2025. The findings reveal that CSEs experience a distinct constellation of occupational stressors, including cumulative trauma exposure, organizational strain, and professional invisibility. Five overarching themes emerged: (1) occupational exposure to trauma and post-traumatic stress; (2) stress, anxiety, and related psychological outcomes; (3) lack of institutional and organizational support; (4) low forensic awareness and diminished role recognition; and (5) protective factors such as resilience, peer support, and humor. Despite recurrent exposure to death, violence, and emotionally charged scenes, many CSEs demonstrate adaptive coping and resilience, though often without formal support structures. The review highlights systemic deficiencies in leadership, debriefing, and psychological monitoring, which exacerbate mental health risks and may impair decision-making quality. Addressing the invisibility of forensic policing through trauma-informed leadership, structured mental health interventions, and enhanced forensic awareness is critical both for workforce sustainability and the reliability of justice processes.

## Introduction

Police work is inherently stressful, characterized by repeated exposure to trauma, high responsibility, and demanding organizational conditions. Research consistently demonstrates that police officers experience elevated risks of post-traumatic stress disorder (PTSD), depression, burnout, and anxiety compared to the general population (Hansson et al., 2017; Ghazinour and Eriksson, 2019; Clarke et al., 2015; Richards et al., 2021). These outcomes are linked to operational stressors such as violence and shift work, but also to structural pressures like inadequate leadership and limited organizational support.

However, this body of research primarily focuses on first responders, such as patrol officers, emergency units, and tactical teams, while other police groups remain understudied. One such group is crime scene examiners (CSEs). Although their technical and procedural roles are well documented, systematic research on their mental health and wellbeing is scarce. This lack of attention is notable because the nature of forensic police work differs markedly from other branches of policing. In this review, the term CSE refer to personal responsible for the examination of crime scenes and the recovery, documentation, and preservation of physical evidence. The professional status of CSEs varies internationally. Crime scene work may be carried out by sworn police officers, civilian forensic technicians, or mixed/hybrid structures with differing police powers, responsibilities, and training backgrounds. To avoid ambiguity with frontline police roles, we use CSE as an umbrella term for all professionals performing crime-scene–focused forensic work, irrespective of their sworn status or organizational placement.

CSEs play a crucial role in criminal investigations by collecting, analyzing, and interpreting physical evidence such as DNA, fingerprints, tool marks, and biological traces (Police Authority). Their task is to reconstruct what happened, how, when, and by whom—often when there are no witnesses or cooperative suspects. Crime scene investigation sits, as Layton (2021, in Measeles, 2023) describes, at the intersection of science, logic, and law. It demands precision, patience, and objectivity under conditions that can be both technically and emotionally challenging (Craven et al., 2022). Operationally, CSEs are no longer merely “second responders”. In serious incidents such as daytime homicides or shootings, they are often deployed alongside patrol units and may be among the first to enter and the last to leave a scene. Their work requires long periods in close proximity to violent or graphic environments, processing bloodstains, human remains, or other forms of physical trauma, while relying heavily on sensory cues such as smell and touch (Ludwig et al., 2012). This prolonged, sensory exposure to death and destruction distinguishes forensic policing from the more immediate, event-based exposures typical of emergency responders. The organizational context compounds these challenges. At the same time, the culture of policing tends to stigmatize emotional vulnerability, reinforcing a tendency to suppress distress or “carry on” (Ghazinour and Eriksson, 2019). These intersecting pressures, sensory, cognitive, and organizational, suggest that CSEs may experience a distinct constellation of occupational stressors, though this has not been systematically examined.

The lack of a comprehensive synthesis of this subgroups' mental health represents a significant gap in understanding police occupational health. Addressing this gap is important because research on CSEs is fragmented, terminologically inconsistent, and dispersed across different disciplines and jurisdictions. Existing studies suggest that CSEs may face forms of stress and trauma exposure that differ from those of frontline responders (Craven et al., 2022; Ludwig et al., 2012), yet these insights have never been systematically mapped or compared. Moreover, little is known about how organizational factors such as role recognition, leadership, or professional status shape CSEs' psychological wellbeing or their ability to cope with cumulative exposure to distressing scenes. A systematic synthesis might contribute to what is currently known, identify conceptual blind spots, and provide a foundation for future empirical and practical developments. Against this background, the present study presents a scoping review to systematically identify and map existing research on the mental health of CSEs. The central research question is: what themes and patterns emerge in existing research on CSEs' mental health and wellbeing? We opted intentionally for a broad research question is because existing studies on CSEs are limited, methodologically dispersed, and often examined in isolation. A wide scoping approach allows us to capture organizational, and operational factors that affect CSEs' wellbeing and resilience, to identify recurring themes as well as neglected areas, and to provide a structured foundation for more targeted research and practical interventions.

The paper proceeds as follows. Firstly, we briefly account for the general literature on police health. Secondly, we outline the methodological scoping review approach. We present the results and discuss the implications for research and practice, where we suggest a that CSEs' mental health should be conceptualized within established (occupational) mental health frameworks more clearly.

## Police mental health

Police officers are repeatedly exposed to operational and organizational stressors that make them vulnerable to a range of mental health problems. Meta-analyses show that police officers experience significantly higher rates of post-traumatic stress disorder (PTSD), depression, anxiety, and burnout than the general population (Syed et al., 2020; Purba and Demou, 2019). Pooled prevalence rates of PTSD among police typically range between 14 and 19 percent—three to four times higher than in the general public (Syed et al., 2020). These findings illustrate the enduring strain that characterizes police work and its potential long-term consequences for both individual wellbeing and organizational performance. For instance, a growing body of research has shown that the occupational strain translates into poor mental health, which in turn, affect police officers' decision-making and use of force (Burke and Mikkelsen, 2005; Kop and Euwema, 2001; Staller et al., 2019).

Research shows that police officers often begin their careers in relatively good psychological condition but experience a gradual decline in wellbeing due to the combined effects of trauma exposure, shift work, disrupted sleep, and organizational pressure (Ghazinour and Eriksson, 2019). Longitudinal evidence suggests that repeated exposure to violent events and irregular hours contributes to cumulative fatigue and stress-related disorders (Violanti et al., 2018). In addition to traumatic incidents, police officers are affected by organizational stressors, including inconsistent leadership, limited staffing, bureaucratic constraints, and inadequate psychological support (Hansson et al., 2017; Pavšič Mrevlje and Erculj, 2021).

Coping and stigma have emerged as central concerns within this field. Many officers rely on avoidance or emotional numbing as coping mechanisms, which may provide short-term relief but exacerbate long-term strain (Pavšič Mrevle, 2021). The occupational culture of policing, often characterized by ideals of toughness and self-reliance, discourages open discussion of mental health issues (Ghazinour and Eriksson, 2019). Officers frequently report fear of being perceived as weak or unfit for duty, leading to a culture of silence around psychological distress. This stigma limits early intervention and contributes to chronic mental health deterioration (Velazquez and Hernandez, 2019). Although this research base has grown substantially, it remains conceptually narrow, with a predominant focus on acute, event-based trauma among frontline responders. Yet many policing roles—including investigative and forensic work—are characterized by chronic, cumulative, and sensory exposure to traumatic material rather than immediate threat (Ferraro and Tobin, 2024; Kelty et al., 2023; Indora et al., 2021; Mountain, 2021). Such exposures may involve repeated contact with human suffering, death, or violence, producing vicarious or complex forms of trauma that are not easily captured by conventional models of post-traumatic stress. In sum, research on police mental health provides robust evidence of widespread psychological strain within the profession, yet the specific mechanisms and manifestations of this strain among CSEs remain poorly understood.

## Method

Because of the relatively small body of research on CSE mental health, we adopted the scoping review approach (Arksey and O'Malley, 2005). According to Arksey and O'Malley (2005), scoping reviews aim to systematically map the breadth and nature of existing research, clarify key concepts, identify knowledge gaps, and synthesize evidence that may be too diverse for traditional systematic review methods. We followed Arksey and O'Malley's (2005) five steps in conducting our scoping review, with the addition that we also followed the Preferred Reporting Items for Systematic Review and Meta-Analysis Protocols Extension for Scoping Review (Tricco et al., 2018), with the exception that no pre-registered protocol was published prior to the review. This decision reflects the exploratory nature of the field and the fragmented evidence base, which required an iterative refinement of search terms and screening criteria during the initial planning stage. However, once the search strategy and inclusion criteria were finalized, no substantive methodological decisions were altered during the review process. In summary, these five steps include (1) identifying the research question; (2) identifying relevant studies; (3) selecting studies based on predefined criteria; (4) charting the data; and (5) collating, summarizing, and reporting the results.

## Search strategy

In collaboration with the university's librarian, we developed a comprehensive search strategy. Since the population of interest goes under many different names, we also did extensive preliminary literature searches to ensure we covered the relevant literature. The search string was divided into two blocks and contained (1) synonyms to “crime scene analyst”, coupled with (2) different types of mental health- and illnesses. The full search string is found below:

“crime scene analyst” OR “crime scene investigator” OR “CSI officer” OR “crime scene technician” OR “crime scene investigation unit” OR “crime scene examiner^*^” OR “forensic crime scene unit” OR “technical crime scene unit” OR “forensic services unit” OR “technical crime scene unit” OR “forensic police officer^*^” OR “forensic investigator” OR “forensic technician” OR “law enforcement forensic^*^” OR “forensic services unit” OR “forensic unit^*^” AND “mental health^*^” OR “mental illness^*^” OR “behavioral health^*^” OR psychosocial^*^ OR ptsd OR anxiety^*^ OR depression^*^ OR burnout^*^ OR distress^*^ OR trauma^*^ OR stress^*^ OR cynicism^*^ OR depersonalization^*^ OR “emotional exhaustion^*^” OR “vicarious trauma” OR emotional^*^ OR wellbeing OR wellbeing OR “quality of life” OR “compassion fatigue^*^” OR “empathy fatigue^*^” OR resilience^*^ OR coping^*^ OR “moral injury”

We searched a total of seven electronic databases using this search string, including PsycINFO, PsycArticles, Criminal Justice Abstracts, Criminal Justice Database, Scopus, Social Sciences Premium Collection, and Web of Science. In addition to this procedure, we conducted extensive back- and forward citation searching from relevant works, as appropriate in scoping review methodology. Google Scholar searches were also used to ensure no relevant works had been left out. The search was performed on the 25^th^ of June 2025.

## Study selection and inclusion/exclusion criteria

To structure our search, we took inspiration from the SPICE model (Setting, Perspective, Intervention, Comparison, and Evaluation). The setting refers to environments where crime scene investigation takes place, such as forensic units, police departments, or fieldwork contexts. The perspective is that of CSEs. The intervention encompasses exposure to potentially traumatic material, occupational stressors, organizational practices, and any psychological or support interventions aimed at addressing mental health. We did not use the comparison condition to structure our search. Finally, the evaluation focuses on mental health outcomes, including but not limited to PTSD, anxiety, depression, burnout, vicarious trauma, resilience, and coping strategies. In addition to these conceptual criteria, we applied several methodological inclusion parameters. We restricted the review to peer-reviewed empirical articles published in English between 2005 and 2025 to ensure accessibility, contemporary relevance, and methodological comparability across studies. We excluded gray literature (e.g., dissertations, reports, conference papers) because such sources vary substantially in methodological rigor, often lack peer review, and could introduce inconsistencies into cross-study comparisons. We acknowledge that this decision may have excluded some potentially relevant insights; however, it allowed us to focus on studies with standardized reporting and quality thresholds.

**Figure 1:**
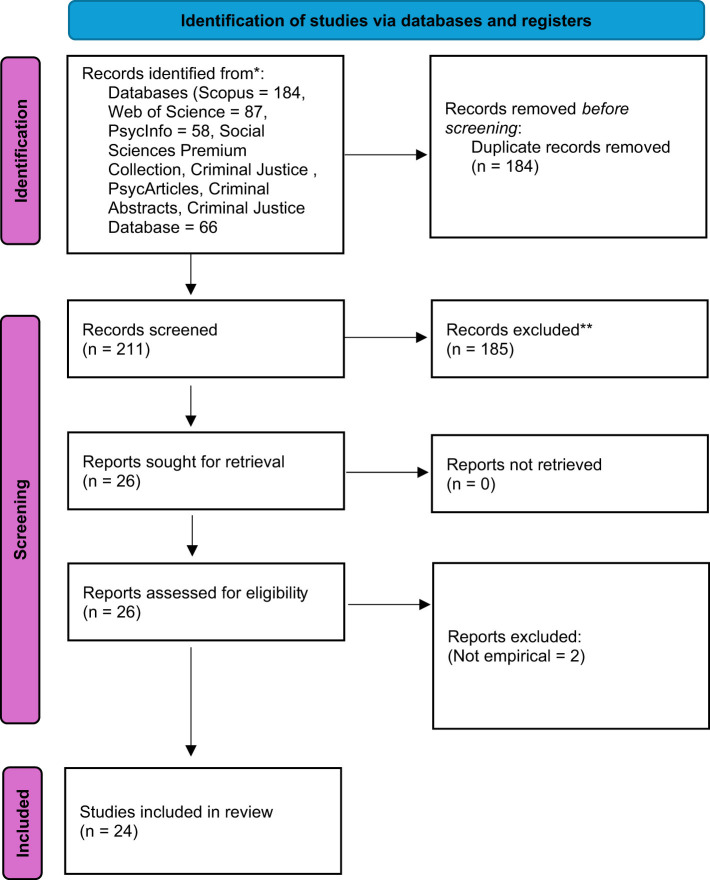
PRISMA flow chart of inclusion process.

According to our SPICE model, in the first round of abstract reviews, the articles had to mention the specific population (CSEs) in relation to their mental health more broadly. Accordingly, we excluded articles if they did not mention the specific population, but instead talk generally about police officers, or specialized units such as IT-forensics. We also excluded articles that did not have a clear mental health scope. Finally, gray litterateur was excluded. The first two authors both reviewed all abstracts independent of one another. Potential disagreements were discussed between the two of them, with the additional plan to include the third author if disagreements were not solved in the first phase; however, this never occurred. After this abstract scanning, the first- and second authors read all articles in full text, again, independent of one another and repeated the process. The full process is illustrated in the flow chart below (Table 1 and Figure 1).

**Table 1:** Overview of included studies.

**Authors**	**Country**	**Aim**	**Method/sample**	**Key findings**
(Adderlay et al. 2012)	UK	To identify the level of increased stress in CSEs	Quantitative method, 12 (*n*)	Results show that CSIs undergo psychosocial stress during the examination of routine crime scenes
(Alexander et al. 2025)	UK	This study explores the lived experiences of CSEs	Qualitative method, 7 (*n*)	Their findings highlight the intense nature of their work. Organizations must prioritize a positive mental health culture in the workplace.
(Almazrouei et al. 2021)	USA	To examine the sources of stress among CSEs.	Mixed methods, 41 (*n*)	Findings showed that CSEs experience high levels of stress due to organizational pressures and lack of support and emotional exposure that can affect their judgement and decision making
(Clarke et al. 2015)	USA	Examines levels of stress related to CSEs' work and the nature of organizational support available to the investigator.	Quantitative method, 28 (*n*)	Findings showed that 63 % of the CSEs reported moderate to high stress after critical incidents and inadequate organizational support intensified the negative impact of their wellbeing and job performance
(Haim-Nachum and Levy-Gigi 2019)	Israel	The aim of the present study was to examine the interactive effect of exposure and training on generalization learning.	Quantitative method, 82 (*n*)	Key findings showed that exposure to traumatic images impaired participants ability to generalize learned information, but targeted training significantly reduces this negative effect
(Hyman 2005)	Israel	The aim was to investigate the prevalence of secondary traumatic stress symptoms among CSEs	Quantitative method, 244 (*n*)	Higher levels of perceived social support were significantly associated with lower levels of secondary traumatic stress
(Kelty and Gordon 2015)	Australia	To develop a partial psychometric profile of forensic personnel who excel in their role and have been identified by their peers and supervisors as being resilient practitioners	Mixed methods, 19 (*n*)	Kelty and Gordon found that forensic workers showed resilience and did not exhibit burnout despite repeated exposure to traumatic events. Results with attention on why forensic organizations should invest in promoting stress management strategies in their employees
(Kelty et al. 2011)	Australia	The study aimed to identify why some CSEs consistently outperform others	Qualitative, 11 (*n*)	The article discusses the management of the crime scene as a critical issue in the justice process
(Macay-Davies et al. 2020)	USA	To examine and compare job related occupational and psychological stress and traumatic exposure experienced by CSEs	Quantitative method, (sample size not specified)	CSEs reported significantly higher on duty stress, both psychological and physical, compared to sworn police officers
(Pavšič Mrevlje 2016)	Slovenia	To examine copying strategies (approach vs avoidance) stress symptoms and health problems due to exposure to traumatic situations.	Quantitative method, 64 (*n*)	Slovenian CSEs face work related traumatic situations. The most common copying strategy is avoidance. Approach copying was associated with lower psychological stress. Need for targeted support and training are emphasized
(Pavšič Mrevlje 2018)	Slovenia	To explore trauma-related psychological functioning in Slovenian CSEs using Rorschach Performance Assessment system and to identify pattern associated with high levels of PTSD symptoms.	Quantitative method, 64 (*n*)	Slovenian CSEs showed high PTSD symptoms. Avoidance was common. Rorschach indicators revealed impaired cognitive and emotional processing in those most affected by trauma.
(Rosansky et al. 2019)	USA	The aim is to investigate the prevalence of PTSD symptoms among CSEs, particularly how they experience and cope with repeated exposure to traumatic events.	Qualitative method, 17 (*n*)	The study underscores the critical need for organizational mental health support structures.
(Sollie et al. 2019)	Netherlands	To explore how CSEs perceive and cope with the psychological and emotional impact of their work	Qualitative method, based on observations of 5 CSE teams and 35 semi-structured interviews with CSEs	CSEs are exposed to trauma, irregular hours, and high responsibility. They cope using cognitive control, emotional distancing, peer support, and sense making. Mental resilience is shaped by individual traits, team support, and organizational recognition. A supportive team culture and reduced stigma around help-seeking are crucial for well-being.
(Tehrani 2024)	UK	To assess the prevalence in psychological distress in forensic investigators and to evaluate how ongoing psychological surveillance can help detect risk and support resilience	Quantitative method, 2028 (*n*)	Suggested support: Routine psychological screening and risk assessments access to trauma information therapy resilience training. Building organizational support structures to reduce stigma
(Vivona 2013)	USA	To explore how CSEs use humor	Qualitative method, 7 (*n*)	CSEs use humor a s emotional release –dark gallows humor to release emotional tension caused by the constant exposure to trauma
(Vivona 2014)	USA	To explore how CSEs use humor to manage emotional stress and build up cohesion	Qualitative method. 23 CSEs	CSEs use humor to support emotional regulation used during emotional demanding tasks to maintain focus reduce distress and to carry out under pressure
(Craven et al. 2022)	USA	To identify psychological and experimental predictors of successful copying among active CSEs and CSE trainees	Mixed methods study, 75 CSE and 88 CSE trainees	Resilience and emotion regulation were the strongest predictors of successful copying among CSEs, with experienced staff -showing higher self- efficacy, mindfulness and lower stress than trainees. Qualitative interviews revealed that confidence, task- focus, meaning in work, and peer support were central to how CSEs manage trauma and sustain resilience.
(Julian and Kelty 2015)	USA	To identify and discuss key risk factors in the use of forensic science in the criminal justice system by using a holistic and systematic approach that examines the collection and use of forensic evidence from crime scene to court	Mixed methods study, 11 detailed case studies of serious crimes.	The authors identified six major risk factors in forensic processing –from low forensic awareness among first responders to poor communication and understanding from the justice system. The research suggests practical reforms to improve reliability and efficiency
(Levin et al. 2021)	USA	To examine secondary traumatic stress, burnout, and compassion satisfaction among CSEs and to assess the relationship between these outcomes and perceived organizational support	Quantitative method, 419 (*n*)	Field-based forensic staff had higher secondary traumatic stress (STS). Burnout and compassion satisfaction were similar across roles. Perceived organizational support reduced STS and increased wellbeing.
(Rosh and Vivona 2010)	USA	To explore how humor is used by CSEs working regularly in environments of death and tragedy	Qualitative method, 12 (*n*)	Humor functions as an adaptive copying mechanism in the high stress emotionally intense work of CSEs. Black humor and inside jokes are used to process trauma, manage trauma, facilitate social bonding and create coherence among colleagues.
(Salinas and Webb 2015)	USA	To assess the stress levels, anxiety levels and coping mechanisms of CSEs across the state of Texas.	Quantitative method, 76 (*n*)	CSEs showed low stress and anxiety levels. Adaptive coping, especially among women supported wellbeing. Promoting such strategies is recommended.
(Winter 2024)	UK	To better understand how organizational culture and leadership affects stress levels amongst CSEs.	Qualitative method, 75 (*n*)	High resilience among CSEs was linked to lower risk of
(Saari et al. 2020)	Finland	The aim was to investigate the wellbeing of finish CSEs	Mixed methods study, 93 (n)	CSEs experienced emotional exhaustion, psychosocial burden and reduced wellbeing. Clear leadership, social support and perceived organizational factors were identified as protective factors.
(Pertsev and Tychyna 2025)	Romania	The aim was to identify key risk factors, evaluate current prevention and recovery strategies, and examine the effectiveness of current technologies such as virtual reality and augmented reality in reducing stress and PTSD symptoms	Systematic review of existing literature and data on occupational burnout and PTSD among CSEs.	Recent studies show that about 30% of CSIs experience occupational burnout and 9.3% meet diagnostic criteria for PTSD. These findings underscore the need for targeted interventions, including psychological screening.

## Data charting

In the data charting phase, we developed a code sheet with inspiration from Peters et al. (2015). This code included (1) authors, (2) year of publication, (3) country, (4) aims and purposes, (5) study population and sample size, (6) methodology, (7) theoretical frameworks, and (8) key findings. After completing the data charting process, we conducted an inductive qualitative analysis to identify patterns across the included studies. Following Braun and Clarke's (2006) principles of thematic analysis we undertook several analytic steps. First, all charted data were read repeatedly to ensure familiarity. Second, we generated initial codes by systematically reviewing each study's key findings, methodological notes, and reported psychological or organizational outcomes. Third, these codes were iteratively grouped into broader conceptual categories through constant comparison across studies. Finally, the categories were refined into the overarching themes presented in the Results section.

## Results

This scoping review identified 24 international studies published between 2005 and 2025. Eleven of these studies originate from the United Forensic States, four from the United Kingdom, two from Australia, two from Israel, two from Slovenia, one from the Netherlands, one study came from Finland and one from Romania. Ten articles were quantitative, nine were qualitative and five were mixed methods studies. Table 2 shows an overview of the results.

**Table 2:** Review results description.

**Theme**	**Description**	**Focus**
Psychological outcomes	CSEs encounter repeated exposure to violent, graphic, and sensory-intensive scenes (blood, decomposition, death), often for extended durations. Sensory load (smell, touch, proximity) differentiates their trauma exposure from that of frontline police.	Prevalence of secondary traumatic stress; PTSD risk; cumulative exposure; sensory overload; impact on cognitive functioning
Lack of institutional support	Many CSEs report insufficient psychological, supervisory, and organizational support. Support structures are inconsistent and often symbolic rather than functional.	Inadequate debriefing; stigma around help-seeking; lack of structured follow-up; poor leadership responses; perceived organizational indifference.
Organizational and structural stressors	Stress emerges not only from traumatic scenes but also from systemic issues such as workload, time pressure, shift patterns, interdepartmental communication, and forensic backlogs	High caseloads; administrative pressure; role overload; operational tempo; limited resources.
Forensic awareness and role perception	Low forensic awareness in broader policing environments affects CSEs' work recognition, role clarity, and identity. Forensic personnel often report feeling invisible or misunderstood	Miscommunication with frontline officers; lack of understanding of forensic processes; undervaluation of forensic expertise; weak integration into investigative workflows
Protective factors and resilience	Despite stressors, many CSEs demonstrate resilience through experience, training, coping strategies, humor, team cohesion, and meaning-making. Supportive organizational cultures improve well-being	Peer support; gallows humor; experience-based confidence; resilience training; reflective practice; strong team culture.

**Figure 2:**
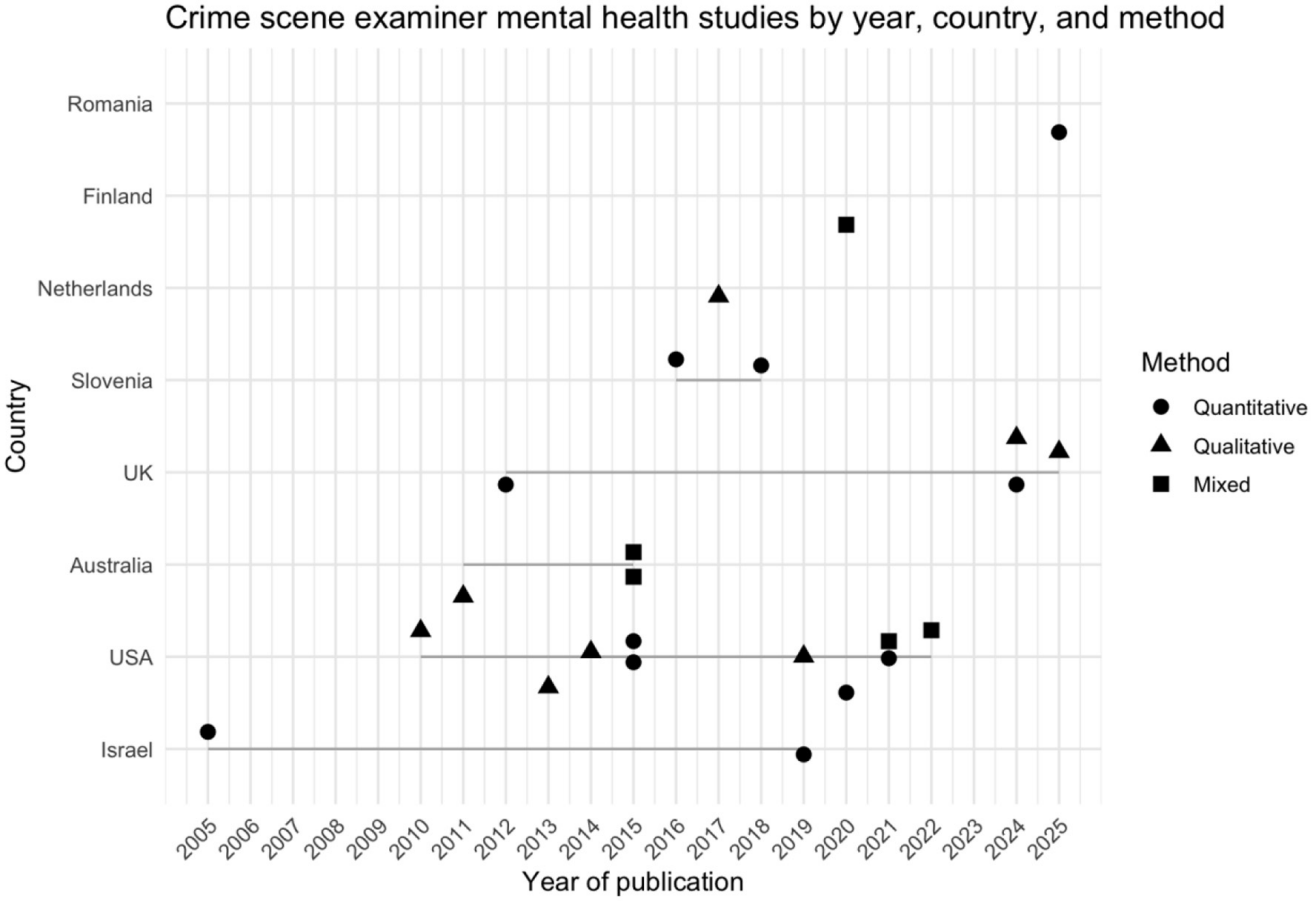
Crime scene examiners' mental health studies across year, country and method between 2005–2025.

The evolution of the literature shows no strong linear trend in terms of publications per year – between one and two studies on CSE mental health are published annually, except for the year 2015 where four studies were published. In terms of method, no clear patterns emerge either. The field is relatively evenly distributed methodologically speaking, with 11 quantitative studies, eight qualitative studies and five mixed method studies. A clear pattern is, however, that the literature on CSE mental health has its strongest base in the Anglo-American countries (USA, UK, and Australia), with no studies from the Global South. These characteristics are visualized below in Figure 2.

Having described the publication trends, methodological distribution, and geographical origins of the included studies, we now turn to the thematic patterns that emerged from our inductive analysis. Across the 24 studies, five overarching themes and subthemes were identified: (1) psychological outcomes; (2) lack of institutional support; (3) organizational and structural stressors; (4) forensic awareness and role perception; and (5) protective factors and resilience. These themes synthesize the psychological, organizational, and cultural dynamics that shape CSEs' mental health across different national contexts. We acknowledge that these themes in part overlap with one another. Each theme is presented in below Table, and subsequently in detail below.

## Psychological outcomes

This theme synthesizes evidence on how repeated exposure to graphic, violent, and sensory-intensive material affects the psychological health of CSEs. Across national contexts, studies consistently show that trauma exposure is a defining and chronic feature of crime scene work, producing a range of mental-health outcomes.

CSEs routinely encounter death, decomposition, blood, and other disturbing stimuli, resulting in sustained psychological strain. (Levin et al. (2021) report elevated levels of stress and emotional exhaustion among forensic practitioners, underscoring the distinct psychological burden associated with crime scene work. Similarly, Alexander et al. (2025) describe how CSEs experience their work as emotionally demanding and mentally depleting, often requiring emotional detachment to maintain functionality. Studies also highlight vicarious trauma and secondary traumatic stress as common reactions to repeated exposure (Salinas and Webb, 2015; Rosansky et al., 2019).

A central contribution comes from Haim-Nachum and Levy-Gigi, 2019, who show that exposure to traumatic images can impair generalization learning—a finding suggesting that trauma affects cognitive functioning even among trained professionals. This introduces a “training paradox”: while training prepares CSEs for distressing scenes, it may simultaneously reveal underlying vulnerabilities triggered by repeated exposure.

Across regions, PTSD symptoms emerge as a major outcome. Rosansky et al. (2019) found that repeated exposure to death and child-related cases strongly predicted PTSD symptom severity. Pavšič Mrevlje's (2016, 2018) Slovenian work similarly documents elevated PTSD scores and impaired emotional processing among trauma-exposed CSEs. Avoidance coping, identified in several studies, appears to reduce short-term distress but contributes to long-term emotional dysregulation and decision-making difficulties (Pavšič Mrevlje, 2018; Hyman, 2005). Larger-scale evidence reinforces these findings. Tehrani (2024), using psychological surveillance data from more than 2,000 UK practitioners, reports high rates of PTSD, anxiety, and burnout, especially among CSEs handling serious crimes. These data emphasize the cumulative nature of trauma in forensic work.

The literature also notes variation by gender and role status. Salinas and Webb, 2015 found that male CSEs reported higher stress and anxiety than female colleagues, while civilian examiners and sworn officers displayed different coping styles. Almazrouei et al. (2021) show that chronic stress may impair forensic judgment and decision-making, indicating consequences not only for wellbeing but for investigative accuracy.

Finally, multiple studies identify psychosocial symptoms such as fatigue, numbness, intrusive imagery, and emotional blunting as common among CSEs (Levin et al., 2021; Pavšič Mrevlje, 2016). These outcomes appear intrinsic to the sensory, cumulative, and prolonged nature of forensic exposure.

Taken together, the literature portrays crime scene examination as an occupation defined by chronic and multi-layered trauma exposure. Mental-health outcomes among CSEs arise not primarily from isolated critical incidents but from the cumulative, sensory, and prolonged nature of repeated exposure, resulting in a constellation of symptoms that include stress, burnout, PTSD, cognitive disruptions, and emotional dysregulation.

## Lack of institutional support

This section examines how organizational structures shape the mental health of CSEs. Across national contexts, the literature consistently points to systemic neglect, inadequate leadership, and entrenched stigma surrounding psychological support.

Multiple studies highlight a pervasive absence of structured wellness programs, debriefing mechanisms, and psychological resources for CSEs. Alexander et al. (2025) and Winter (2024) identified systemic deficiencies in mental health provision for CSEs in the United Kingdom, where civilian CSEs were particularly vulnerable due to their exclusion from established police wellness infrastructures. Leadership failures were shown to foster a culture of silence in which officers avoided discussing distress, reinforcing stigma around help-seeking and generating feelings of isolation and powerlessness. Similar findings emerged in Pavšič Mrevlje (2016), who documented elevated stress linked to the lack of institutional recognition and support.

Even where psychological services exist, utilization remains low due to persistent cultural and occupational barriers Winter (2024). In the United States, Macay-Davies et al. (2020) found that civilian forensic technicians had both lower awareness of mental health services and higher levels of job-related stress compared to sworn police officers. Organizational hierarchies appear to mediate access to support, leaving civilian staff more exposed to chronic strain. Almazrouei et al. (2021) similarly identified organizational pressure, insufficient support, and cumulative emotional exposure as primary stressors that may impair judgment and decision-making in forensic practice. Complementary evidence from Clarke et al. (2015) shows that inadequate organizational response after critical incidents amplifies the negative impact of occupational stress on both wellbeing and performance. Gender dynamics also shape access to institutional and informal support. Almazrouei et al. (2021) found that female CSEs reported greater emotional strain and more limited access to peer support compared to their male counterparts, while Salinas and Webb (2015) observed that women more often adopted adaptive coping strategies associated with lower stress and anxiety. Together, these studies reveal that organizational neglect interacts with gendered expectations and professional hierarchies to produce unequal patterns of psychological risk.

Overall, the literature demonstrates that the absence of institutional care is not a passive omission but an active structural condition. Organizational inertia, stigma, and inequitable access to support mechanisms compound the psychological toll of forensic work, underscoring that resilience cannot be understood solely as an individual trait—it is a function of institutional responsibility.

## Organizational and structural stressors

This section examines how organizational and structural arrangements create chronic psychological strain among CSEs. The literature consistently demonstrates that stress within forensic policing is not incidental but structurally embedded in workload systems, leadership culture, and institutional identity. Key mechanisms include high responsibility combined with low control, poor organizational recognition, and the marginal status of civilian CSEs within police institutions.

Multiple studies identify organizational conditions as primary stressors for CSEs. Adderlay et al. (2012) found that even routine crime scene work—typically not categorized as traumatic—produced significant psychological strain. The constant pressure to achieve rapid yet flawless documentation, coupled with awareness that minor errors can have major legal consequences, generated chronic stress. These demands were particularly pronounced in the United Kingdom, where CSEs are often civilian employees without the police training or occupational support systems available to sworn officers. In contrast, CSEs in the United States and Canada typically begin their careers as police officers, receiving early exposure to stress-management and resilience training at the police academy and having access to the same wellness infrastructures as frontline staff. The UK-based CSEs, lacking such institutional scaffolding, described feeling unprepared when confronted with hostile or distressed victims and frustrated by public expectations influenced by the “CSI effect”—a perception that forensic analysis can instantly solve crimes. Unrealistic expectations, high accountability, and low control over outcomes combined to create a state of anticipatory stress that emerged even before entering a crime scene. As Adderlay et al. (2012) observed, this occupational tension threatens not only individual wellbeing but also the reliability of evidence recovery and, ultimately, case outcomes.

Leadership culture and organizational neglect are recurrent sources of psychological strain. Alexander et al. (2025) reported that CSEs often felt dehumanized—treated as “numbers on a pay-check” rather than valued professionals—reflecting a managerial assumption that forensic staffs are inherently resilient and unaffected by trauma. Winter (2024) similarly demonstrated that leadership behaviors are decisive in shaping resilience: poor supervision, limited decompression time, and inadequate recognition function as independent stressors, while caring and engaged leadership mitigates these effects. Many CSEs described feeling powerless to influence workload or recovery periods, deepening exhaustion and eroding organizational trust. The cumulative impact of these factors is what several authors describe as ongoing stress—a persistent strain that pervades even routine investigations such as burglaries or thefts.

Across studies, avoidance coping emerged as a dominant strategy. Alexander et al. (2025) observed that CSEs frequently compartmentalize emotional responses—“putting it in a box”—as a means of functional survival. While such detachment enables operational continuity, it also normalizes emotional suppression and inhibits long-term psychological processing. Clarke et al. (2015) reported that CSEs experienced moderate to high stress following critical incidents, with insufficient organizational support exacerbating symptoms. Participants commonly expressed frustration at the lack of recognition for their work, reinforcing perceptions of institutional neglect. Almazrouei et al. (2021) linked similar organizational stressors—such as workload backlogs, poor supervision, and limited managerial feedback—to diminished judgment quality and decision-making accuracy, suggesting that psychological strain may compromise evidential reliability.

The relationship between organizational structure and resilience is similarly mediated by recognition and professional culture. Kelty and Gordon, 2015 emphasized that resilience is not a fixed individual trait but dependent on institutional context: CSEs can sustain performance under stress only when supported by fair leadership and meaningful acknowledgment of their work. Earlier research by Kelty et al. (2011) identified professional identity and sense of purpose as protective factors, while Julian and Kelty (2015) highlighted systemic risks such as low forensic awareness among first responders and weak interdepartmental communication that undermine morale and investigative effectiveness. These findings converge with Levin et al. (2021), who showed that perceived organizational support significantly reduces secondary traumatic stress and burnout. Sollie et al. (2019) similarly found that team cohesion, peer recognition, and positive workplace culture serve as buffers against the cumulative effects of heavy caseloads and irregular hours. Gender dynamics also intersect with organizational factors: Salinas and Webb (2015) found that female CSEs more often adopted adaptive coping strategies associated with lower stress, yet Almazrouei et al. (2021) observed that women reported less access to informal peer support, illustrating how gendered organizational norms can unevenly distribute resilience resources.

While most studies highlight strain, some also reveal conditions that sustain satisfaction and commitment. Saari et al. (2020) found that Finnish CSEs reported high organizational commitment and job satisfaction (89%), citing collegial cooperation, task autonomy, and the meaningfulness of contributing to justice as central to their wellbeing. These findings suggest that the psychosocial impact of forensic policing is not universally negative but contingent upon the organization's ability to foster autonomy, teamwork, and recognition. Even within high-stress environments, supportive leadership and collective efficacy can transform demanding work into a source of professional fulfillment.

### Forensic awareness and role perception

Within the broader category of organizational and structural stressors, several studies highlighted a specific mechanism the literature refers to as “forensic awareness”—that is, the degree to which CSEs' expertise is recognized, understood, and integrated into investigative processes. We therefore treat forensic awareness and role perception as a subtheme of organizational stressors, reflecting its function as a structural rather than standalone psychological mechanism. This section examines how the low institutional visibility of CSEs produces structural neglect, professional marginalization, and heightened psychological strain. The literature consistently shows that weak forensic awareness undermines both occupational wellbeing and the reliability of justice outcomes.

A consistent finding across studies is that the invisibility of CSEs within police organizations and the broader justice system is a major source of occupational stress. The absence of understanding about the nature of forensic work often results in limited recognition, inadequate integration into investigative decision-making, and underinvestment in wellness structures. This lack of institutional acknowledgment erodes professional identity and increases psychological strain. Julian and Kelty's (2015) seminal 5 year study, Forensic Science as Risky Business, examined 11 major crime cases and identified six systemic risk factors across the investigative chain. Their findings demonstrated that “good forensic science begins at the crime scene,” yet low forensic awareness among first responders and supervisors led to errors in evidence preservation and interpretation. Similarly, their large-scale review of 41 police districts in England and Wales revealed that insufficient understanding of forensic processes among operational staff was a major source of inefficiency. CSEs were often perceived merely as evidence collectors or technical support rather than as integral investigative professionals. Julian and Kelty concluded that the quality of forensic output was directly dependent on whether CSEs were institutionally supported and professionally recognized. Despite the significance of this construct, no standardized international instrument currently exists to measure forensic awareness.

Subsequent research confirms these patterns. Jeanguenat and Dror (2018) argued that debates on forensic reliability overemphasize cognitive bias while neglecting organizational factors—particularly the institutional undervaluation of forensic expertise. Winter (2024) similarly reported that CSEs in the UK were excluded from wellness programs available to other police staff, interpreting this exclusion as a symptom of low forensic awareness at leadership level. Alexander et al. (2025) found that UK CSEs described their work as emotionally taxing yet institutionally misunderstood; despite frequent exposure to trauma, they received little recognition or psychological support. These findings converge on the idea that forensic invisibility operates both as a cultural and structural condition: when leadership fails to grasp the emotional and operational complexity of forensic work, neglect becomes normalized.

The consequences of low forensic awareness are further complicated by gender and civilian status. Sollie et al. (2019) found that Dutch CSEs without police academy training—often civilian employees—experienced feelings of exclusion and “outsider” identity, which weakened their access to informal peer support networks and reduced operational effectiveness. In the US, Macay-Davies et al. (2020) reported that civilian CSEs experienced higher psychological stress than sworn officers due to lower recognition, limited status, and exclusion from police wellness systems. Across studies, this status-based marginalization is not merely symbolic; it has tangible consequences for both individual resilience and organizational function.

At the cultural level, low forensic awareness perpetuates stigma around help-seeking and inhibits institutional reform. Multiple studies (Julian and Kelty, 2015; Jeanguenat and Dror, 2018; Winter, 2024; Rosansky et al., 2019) emphasize that the unique cognitive and emotional demands of forensic work remain poorly understood by leadership structures. This lack of understanding correlates with greater psychological strain, symptoms of post-traumatic stress, and in some cases, early career attrition (Rosansky et al., 2019). The invisibility of CSEs within the policing hierarchy thus represents not only a failure of organizational empathy but a structural weakness in the justice system itself: when the actors responsible for securing and interpreting evidence are marginalized, both staff welfare and evidential reliability are compromised.

## Protective factors and resilience

This section examines the factors that support wellbeing and resilience among CSEs. The literature conceptualizes resilience not as a static personal trait but as a dynamic process shaped by individual characteristics, social networks, and organizational structures. Protective factors operate at both the personal and institutional level, enabling CSEs to sustain performance and psychological health despite chronic exposure to trauma and operational pressure.

### Individual-level attributes and cognitive mechanisms

Numerous studies identify personal attributes linked to sustained wellbeing among CSEs. Traits such as mindfulness, optimism, emotion regulation, self-esteem, and a strong internal locus of control have been associated with reduced psychological distress and higher professional satisfaction. The importance of resilience for coping has been most systematically demonstrated in a series of studies by Kelty et al. (2011) and Kelty et al. (2024) identified seven attributes consistently linked to high performance—professional knowledge, communication, composure under pressure, life experience, approach to work, self-discipline, and overall professionalism—all of which align closely with resilience factors.

Kelty and Gordon (2015) compared the psychometric profiles of 19 high-performing CSEs with those of police officers, students, and the general population. CSEs displayed higher critical-thinking ability and lower depression and anxiety than police officers, while showing similar stress resilience to the general population. These findings suggest that the most effective CSEs possess measurable psychological attributes that can be identified during recruitment and developed through training. Kelty and Gordon argue that “hiring well” is a form of preventive intervention: selecting and supporting individuals with high resilience potential reduces operational errors and enhances both wellbeing and evidential reliability. Adequate managerial resources and clear procedural guidance were also highlighted as critical contextual enablers of resilience.

Craven et al. (2022) provide one of the most comprehensive examinations of coping and resilience in forensic policing. Drawing on Lazarus and Folkeman (1984) transactional theory of stress, they conceptualize coping as a dynamic interaction between individual appraisal and environmental demands. Their findings show that successful coping depends not on fixed personality traits but on the ability to mobilize multiple strategies—problem-focused, emotion-focused, and meaning-focused—when facing traumatic exposure. Importantly, resilience was found to develop over the career span, supported by external factors such as peer recognition, leadership, and institutional culture. These findings align with Reich et al. (2010) view of resilience as an outcome of effective coping rather than an innate disposition.

### Social and organizational enablers

Protective factors are not limited to individual capacities. Mentorship, structured induction programs (e.g., shadowing experienced CSEs), and sustained peer and supervisory support have all been linked to long-term adaptation and reduced burnout (Craven et al., 2022; Pavšič Mrevlje, 2016). Salinas and Webb (2015) observed that adaptive coping strategies, particularly among women, were associated with lower stress and greater wellbeing. Hyman (2005) further demonstrated that perceived social support significantly reduces secondary traumatic stress among CSEs. Alexander et al. (2025) emphasize that positive organizational mental-health cultures and transparent communication are essential for maintaining resilience, while Levin et al. (2021) found that perceived organizational support can substantially improve wellbeing across forensic units. Winter (2024) and Tehrani (2024) both argue for formalized resilience training and trauma-informed leadership practices as necessary components of sustainable forensic workforces. These interventions, they suggest, should not only target individual coping skills but also address systemic issues such as workload management, recognition, and inclusion in wellness programs.

### Cultural and symbolic coping

A distinct strand of research highlights humor and meaning-making as central to resilience within forensic culture. Unlike individual emotion-management strategies, humor in this context operates as a cultural and symbolic coping mechanism because it is embedded in shared norms, collective identity, and tacit rules that structure how CSEs interpret and respond to trauma.

Rosh and Vivona (2010), in *Mirth and Murder*, showed how humor provides CSEs with emotional distance from the violence and tragedy they encounter, functioning both as a coping mechanism and a form of professional boundary work. Similarly, Vivona (2013, 2014) documented that humor among CSEs is socially regulated: acceptable in certain contexts as a means of tension release and solidarity but deliberately withheld in moments of grief or respect. This selective use of humor reflects professional judgment and serves as an indicator of group cohesion and moral boundaries. Humor, when appropriately used, thus reinforces emotional regulation, team connectedness, and identity as coping resources that sustain resilience.

Sollie et al. (2019) add that resilience among Dutch CSEs is expressed through a blend of structured routines, humor, and emotional distancing—everyday strategies that allow officers to maintain functionality without emotional collapse. The sense of contributing to justice and helping victims was repeatedly identified as a profound meaning-making mechanism that sustains motivation even in distressing conditions. This aligns with (Craven et al. 2022), who argue that resilience should be cultivated not only in the field but at the entry level, through recruitment, mentorship, and reflective practice.

## Discussion

This review identified a range of patterns shaping the mental health of CSEs. First, CSEs experience substantial psychological strain linked to chronic, sensory-intensive, and cumulative exposure to traumatic material, including elevated levels of stress, secondary traumatic stress, and PTSD symptoms. Second, the literature shows consistent deficits in institutional and supervisory support, with limited access to structured debriefing, mental-health resources, or protected recovery time. Third, organizational and structural stressors such as workload pressure, time constraints, and weak integration into investigative processes further compound these risks. Fourth, low forensic awareness and limited recognition of CSE expertise contribute to feelings of invisibility and marginalization, shaping both wellbeing and professional identity. Finally, several studies highlight protective factors, including humor, team cohesion, experience-based confidence, and meaning-making, which operate as resources that support resilience. Together, these findings portray CSE wellbeing as the outcome of interacting trauma exposures, organizational conditions, and cultural practices. In the discussion, we focus on discussing the organizational conditions and how this interacts with forensic invisibility. We then move on to discuss the theoretical implications, and end with limitations and conclusions.

## Organizational conditions and forensic invisibility

This review exposes a paradox at the core of forensic policing. CSEs manage some of the most decisive components of criminal justice including recovering, preserving, and interpreting evidence (Kelty et al., 2023; Ludwig et al., 2012). Yet, their professional identity and well-being remain largely invisible. This invisibility reflects an epistemic hierarchy within policing that privileges visible enforcement over analytical and interpretive labor (Crispino et al., 2015; Raymond and Julian, 2015). CSEs thus occupy a position central to evidential truth but peripheral to organizational authority.

The effects of this invisibility are structural as much as cultural. Organizationally, it translates into limited leadership attention, scarce professional development, and exclusion from wellness systems (Almazrouei et al., 2021). Culturally, it reproduces the notion that forensic work is technical rather than investigative (Chowdhury, 2021), diminishing its symbolic capital within the police institution. The recurrent finding of low “forensic awareness” across the literature illustrates how credibility and authority are distributed (Winter, 2024; Rosansky et al., 2019). When first responders, supervisors, or prosecutors misunderstand the epistemic complexity of forensic work, they perpetuate an institutional order that values control over comprehension. Limited forensic awareness and weak institutional support create conditions under which psychological strain can directly affect operational reliability (Kelty et al., 2023). Chronic exposure to trauma, combined with unrealistic performance demands, elevates the risk of error in evidence handling and interpretation. (Kelty et al. 2011; 2023; 2015; 2023) have, for over a decade, explored what constitutes “high-performing” CSEs in light of the wide variation of competence of CSEs. Naturally, given that crime scene investigation is intellectually challenging, they find that cognitive capacity is critical. Importantly, they also note that CSEs face serious risks of becoming burnt out. In turn, being burned out impair cognitive ability, thus reducing CSEs' problem-solving ability (Kelty et al., 2023). Kelty et al. (2023) describes this in detail and specify how for instance detectives need to be wary about specimen quality that has been collected at a crime scene if the CSE in question is a low performer.

Invisibility and CSEs' organizational conditions, in this sense, becomes operational, as it undermines both wellbeing and the accuracy of justice outcomes. These organizational weaknesses pose direct risks to evidentiary integrity, as the cognitive demands of crime-scene work mean that even small impairments may propagate through the investigative process and affect downstream decision-making across the justice chain. Protecting CSEs' mental health is therefore both an ethical obligation and a prerequisite for epistemic integrity within the criminal process.

## Theoretical implications

Leadership style, peer support, and access to decompression time are well-established predictors of long-term wellbeing in high-strain occupations (Johnson et al., 2005) and can be understood through an institutional or systems-theoretical lens (Eriksson et al., 2018). However, the reviewed literature shows that organizational cultures in forensic policing often transform endurance into a moral ideal, rewarding those who suppress vulnerability. In such contexts, the normalization of suffering converts resilience into an organizational technology—an implicit expectation that individuals absorb systemic stress. Coping, in this sense, becomes synonymous with compliance. Resilience should instead be conceptualized as an institutional achievement emerging from collective practices that distribute emotional risk, facilitate reflection, and enable recovery.

Patterns in gender and employment status further demonstrate how invisibility operates as a layered structure. Female CSEs frequently report greater psychosocial strain and reduced access to informal peer networks, reflecting gendered patterns of recognition and emotional labor (Almazrouei et al., 2021; Alexander et al., 2025). Civilian CSEs experience parallel forms of marginalization, lacking both authority and a secure sense of organizational belonging (Sollie et al., 2019; Winter, 2024). Invisibility is therefore not uniform but stratified, producing differentiated exposure to stress and uneven access to organizational support.

Reframing invisibility in this way moves the discussion beyond descriptive accounts of stress toward a broader theorization of epistemic marginality in policing. CSEs' experiences reveal how hierarchies of knowledge within police organizations shape not only health outcomes but also investigative performance (Kelty et al., 2023). As long as its practitioners remain symbolically subordinate, policing will continue to rely upon a form of cognitive labor it fails to fully value. Addressing this invisibility thus requires an epistemic and cultural shift that positions forensic expertise as integral to investigative legitimacy.

The imbalance that emerges from this review—high emotional, cognitive, and temporal demands combined with minimal structural resources—aligns closely with the Job Demands–Resources (JD–R) model (Bakker and Demerouti, 2007). Within this framework, job demands are aspects of work that require sustained effort and are associated with psychological or physiological costs, while job resources are elements that help achieve work goals, reduce demands, or stimulate development. When demands chronically outweigh resources, the risk of burnout and impaired performance increases; when resources are sufficient, they promote engagement, learning, and resilience. Building on Ungar's (2013) social-ecological account of resilience, particularly his principle that nurture trumps nature in navigating adversity, resilience among CSEs should be understood primarily as a property of environments that enable access to culturally and professionally meaningful resources (such as leadership empathy, protected decompression time, peer recognition, and involvement in investigative decision-making). Ungar's (2013) framework also clarifies that positive adaptation can coexist with symptoms: purpose, cohesion, and professional efficacy may remain intact even in the presence of distress. Importantly, because the value of any resource is context-dependent, the same form of support (such as peer assistance) may offer protection to some practitioners but not to others, depending on their role, gender, or civilian status.

To integrate these insights, we specify epistemic invisibility—the institutional devaluation of forensic knowledge—as a meta-level resource deficit. Invisibility weakens the relationship between organizational resources and wellbeing before individuals can benefit from the supports normally described by the JD–R (Bakker and Demerouti, 2007) and ecological resilience frameworks (Ungar, 2013). This generates a clear research agenda. Future studies could examine whether epistemic invisibility functions as a moderating factor that attenuates the positive effects of organizational resources on health and performance outcomes. This would allow researchers to test whether environmental interventions, such as trauma-informed leadership, structured debriefings or the formal inclusion of CSEs in investigative decision-making, produce greater improvements than interventions focused solely on individual coping skills. In short, integrating JD–R with Ungar (2013) reframes resilience in forensic policing from a matter of personal endurance to one of institutional design, making it measurable, comparable across contexts, and directly actionable.

## Limitations

This review has several limitations. First, the overall evidence base on CSE mental health remains small and geographically concentrated in Anglo-American contexts, which limits the generalizability of the findings. Second, methodological heterogeneity across studies and the absence of a formal quality appraisal (consistent with the scoping review approach; Arksey and O'Malley, 2005) restrict the ability to compare outcomes or evaluate the robustness of individual studies. Third, the review was limited to English-language, peer-reviewed publications, which may have excluded relevant insights from non-English-speaking forensic communities or gray literature. Fourth, variation in role definitions and forensic structures across jurisdictions complicates the synthesis of findings. Finally, because the included studies are primarily cross-sectional and descriptive, causal inferences about the relationship between trauma exposure, organizational conditions, and mental-health outcomes cannot be drawn.

## Conclusions and contributions

This review contributes to several ongoing debates in policing research and advances the literature in ways that extend beyond the dominant focus on frontline police officers. First, it demonstrates that CSEs experience a qualitatively different pattern of occupational stressors, characterized by chronic, cumulative, and sensory-intensive exposure to traumatic material, underscoring that existing models and research derived from frontline policing (Syed et al., 2020) cannot simply be mapped onto forensic work. By foregrounding these distinct exposure profiles, the review broadens the scope of police health research and establishes CSEs as a critical but previously overlooked occupational group.

Second, the review conceptualizes forensic policing as a domain where epistemic hierarchies within police organizations become visible. This reframes forensic work not merely as technical support but as a site where knowledge, authority, and institutional recognition intersect. In doing so, the review moves beyond traditional accounts of police stress to show how epistemic invisibility functions as a structural condition that shapes both health outcomes and the evidentiary quality of the justice process.

Third, the review links occupational health directly to evidentiary reliability. By demonstrating that cumulative trauma, burnout, and organizational neglect can impair cognitive performance and decision-making, it reframes wellbeing as a prerequisite for trustworthy forensic knowledge rather than a secondary welfare concern. This perspective is largely absent from frontline-focused research, which typically centers safety, trauma, and public contact rather than evidentiary integrity.

Finally, the review reconceptualizes resilience as a collective and institutional property, not merely an individual trait. Integrating insights from the Job Demands–Resources model and social-ecological theories of resilience (Ungar, 2013), it specifies how organizational conditions—including leadership, recognition, and workflow design—shape CSEs' capacity to cope with high-stakes investigative work. This theoretical integration offers a framework that is directly transferable to comparative policing research while highlighting the unique pressures of forensic roles. Taken together, these contributions position forensic invisibility not only as an occupational or psychological concern but as a structural vulnerability with implications for the accuracy and legitimacy of contemporary policing. Recognizing and addressing this invisibility is central to maintaining both the integrity of forensic evidence and the broader credibility of the criminal justice system.
